# Recent advances in developing high-performance organic hole transporting materials for inverted perovskite solar cells

**DOI:** 10.1007/s12200-022-00050-3

**Published:** 2022-11-17

**Authors:** Xianglang Sun, Zonglong Zhu, Zhong’an Li

**Affiliations:** 1 Key Laboratory for Material Chemistry of Energy Conversion and Storage, Ministry of Education, School of Chemistry and Chemical Engineering, Huazhong University of Science and Technology, Wuhan, 430074 China; 2 Department of Chemistry, City University of Hong Kong, Kowloon, 999077 Hong Kong China

**Keywords:** Inverted perovskite solar cells, High-performance, Hole transporting materials, Polymer semiconductors, Self-assembled monolayer

## Abstract

Inverted perovskite solar cells (PVSCs) have recently made exciting progress, showing high power conversion efficiencies (PCEs) of 25% in single-junction devices and 30.5% in silicon/perovskite tandem devices. The hole transporting material (HTM) in an inverted PVSC plays an important role in determining the device performance, since it not only extracts/transports holes but also affects the growth and crystallization of perovskite film. Currently, polymer and self-assembled monolayer (SAM) have been considered as two types of most promising HTM candidates for inverted PVSCs owing to their high PCEs, high stability and adaptability to large area devices. In this review, recent encouraging progress of high-performance polymer and SAM-based HTMs is systematically reviewed and summarized, including molecular design strategies and the correlation between molecular structure and device performance. We hope this review can inspire further innovative development of HTMs for wide applications in highly efficient and stable inverted PVSCs and the tandem devices.

**Graphical Abstract:**

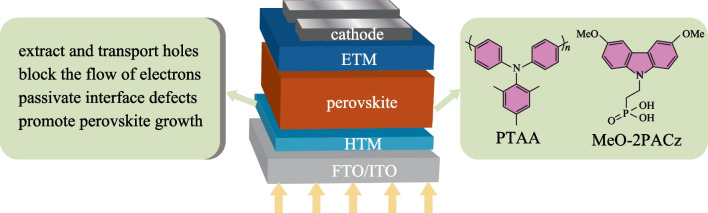

## Introduction

Photovoltaics (PV) holds the key position in the renewable energy field, and has been considered as the most suitable solution both to serious environmental pollution problems and to continuously growing energy demands [1]. Nowadays, silicon solar cells (first-generation photovoltaic technology) still dominate the photovoltaic market due to high efficiency and high device stability, despite having disadvantages such as high cost, inflexibility, and opacity, which limit their future development prospects [2, 3]. Fortunately, perovskites have been explored as one of the most promising photovoltaic materials, due to their advantages of abundance in nature of raw materials, solution processability, remarkable optoelectronic properties, and so on [4–7]. In the past decade, perovskite solar cells (PVSCs) have undoubtedly become the super star in the PV field, and now the certified power conversion efficiency (PCE) has reached a very high level of 25.7%, which is comparable to that of crystalline silicon solar cells [8, 9].

**Fig. 1:**
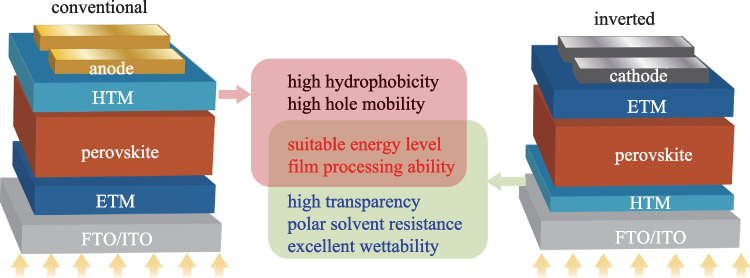
Materials requirements for developing high-performance HTMs in conventional and inverted PVSCs. *ITO* indium-tin oxide, *FTO* fluorine doped tin oxide

Like most of organic optoelectronic devices, PVSCs exhibit multilayer device structure, in which the photo-absorbing layer (perovskite layer) is sandwiched between two charge transporting layers (CTLs), i.e., hole transporting layer (HTL) and electron transporting layer (ETL), as shown in Fig. 1 [10]. According to the position of HTL and ETL, PVSCs can be mainly divided into two common device structures: conventional (n-i-p) and inverted (p-i-n) structure. Compared to conventional PVSCs, inverted PVSCs show several attractive advantages, such as low-temperature fabrication process, high device stability, negligible hysteresis and excellent compatibility with flexible and tandem devices. However, for a long time their device PCEs have lagged behind those of the conventional counterpart [11–17]. Excitingly, the record PCE of inverted PVSCs has recently broken the 25% efficiency barrier [18], thereby strongly suggesting the extraordinary potential for future practical applications.

In general, HTL can perform two essential functions, i.e., extracting/transporting holes and blocking the flow of electrons to the anode, and these behaviors require the hole transport materials (HTMs) to exhibit suitable highest occupied molecular orbital (HOMO) and lowest unoccupied molecular orbital (LUMO) energy levels, and efficient hole extraction/transport ability [19–22]. Due to the different positions of the HTL in conventional and inverted PVSCs, some different specific HTM properties are required (Fig. 1). For conventional PVSCs, high hydrophobicity is required since the HTL is atop the perovskite layer, and can serve as the effective protecting layer for perovskites from moisture exposure. However, HTM is deposited first before the perovskite layer during the fabrication of inverted PVSCs, so it needs suitable surface wettability to act as the substrate for subsequent deposition and crystallization of the perovskite [23–28]. In addition, the transmittance of HTM in inverted PVSCs also affects the light absorption efficiency of the perovskite layer since sunlight needs to pass through the HTL first [29]. Excellent film processing ability as well as high thermal, chemical and morphological stabilities are the basic requirements for HTMs in both conventional and inverted devices [30]. It is worth noting that the HTL thickness in inverted devices is always thin, so the requirement of hole mobility is not as high as that for conventional devices [31].

For a long time, polymer HTMs have been dominant in construction of inverted PVSCs mainly because of their excellent resistance to perovskite precursors, while organic small molecule HTMs have been less used due to two facts: (1) the resulting HTLs would be often damaged by the polar solvents in perovskite precursor solution; (2) the device performance lags far behind that with polymer HTMs. However, this situation has been changed with the recent emergence of small molecule HTMs based on self-assembled monolayer (SAM), which has shown impressive improvement in the device performance, especially in tandem devices [32, 33]. Considering the rapid developments of polymer and SAM-based HTMs recently achieved for inverted PVSCs, a review is presented here to summarize their advances with the purpose of providing researchers with better guidance for further advancing this field.

## Polymer HTMs in inverted PVSCs

**Fig. 2:**
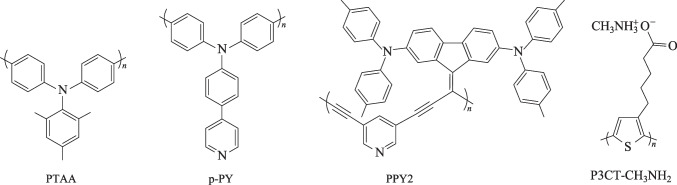
Chemical structure of representative polymer HTMs for efficient inverted PVSCs

Polymer semiconductors exhibit distinct advantages compared to small molecule semiconductors, such as excellent film processing ability, high heat resistance, excellent mechanical properties and compatibility with the large scale roll-to-roll printing technique, which has been widely used in production of organic light-emitting diodes (OLEDs) [34], organic field-effect transistors (OFETs) [35, 36] and organic photovoltaics (OPV) [37, 38], and so on [39, 40]. The encouraging progress of p-type polymer semiconductors therefore offer many good material candidates and molecular design experiences for polymer HTMs, leading to encouraging device performance improvement for both conventional and inverted PVSCs [30, 41–43]. For example, the well-known polymer semiconductor, **P3HT**, has enabled a high PCE of 23.3% when serving as a dopant-free HTM for conventional PVSCs [41]. In this section, we will focus on the latest development of representative polymer HTMs (Fig. 2) in achieving high-performance inverted PVSCs.

**Fig. 3:**
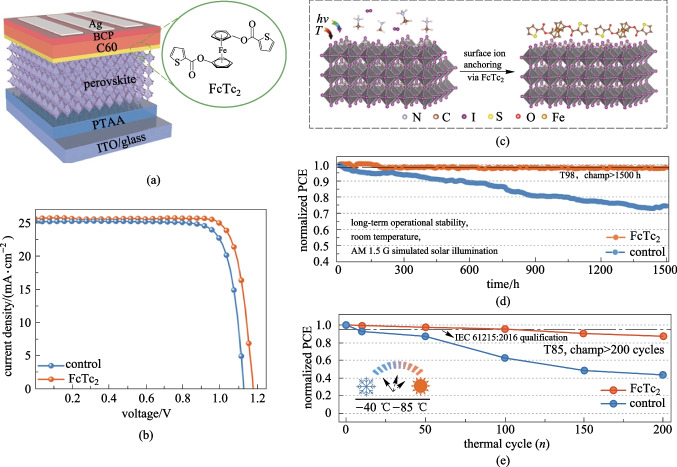
**a** Device structure of inverted PVSCs based on FcTc_2_ as the interface functionalization material. **b** *J*–*V* curves of the champion devices with and without FcTc_2_. **c** Schematic illustration of the stabilization of surface ions by FcTc_2_. **d** Normalized PCE of the unencapsulated PVSCs with or without FcTc_2_ measured at MPP under continuous 1-sun illumination in an N_2_ atmosphere and at room temperature. **e** Normalized PCE of encapsulated PVSCs with or without FcTc_2_ measured in 85% relative humidity and 85 °C in the dark. Reprinted with permission from Ref. [18]. Copyright 2022, The American Association for the Advancement of Science

Poly(bis(4-phenyl) (2,4,6-trimethylphenyl) amine (**PTAA**) (Fig. 2) is the most commonly used HTM for inverted PVSCs and currently holds the record PCE. **PTAA** exhibits advantages of good optical transparency, suitable HOMO energy level (− 5.20 eV), high hole mobility (3–6 × 10^−4^ cm^2^/(V⋅s)) [44, 45], and suitable hydrophobic surface that can regulate the crystalline grain growth of perovskite films [25]. In general, PCEs around 22% can be achieved for **PTAA**-based inverted PVSCs. However, based on **PTAA** HTM, Zhu et al. recently successfully fabricated inverted PVSCs (Fig. 3a) with a record PCE of 25.0% (certified 24.3%, Fig. 3b) with a high open-circuit voltage (*V*_OC_) of 1.18 V, a short-circuit current density (*J*_SC_) of 25.68 mA/cm^2^ and a fill factor (FF) of 82.32% [18]. Such significant performance enhancement was attributed to the introduction of an organometallic compound, ferrocenyl-bis-thiophene-2-carboxylate (FcTc_2_, Fig. 3a), as the interfacial layer between the perovskite layer and ETL. This compound not only accelerates the interfacial electron transfer through the electron-rich and electron-delocalized ferrocene units, but also reduces the surface trap states of perovskites and further stabilizes the surface via strong chemical Pb–O binding (Fig. 3c). Moreover, the FcTc_2_-functionalization can also enable amazing long-term device stability; over 98% of initial PCE can remain after continuously operating at the maximum power point (MPP) for 1500 h under simulated AM1.5 illumination, and the device has successfully passed the international standards for mature photovoltaics (IEC61215:2016) (Fig. 3d, e).

**Fig. 4:**
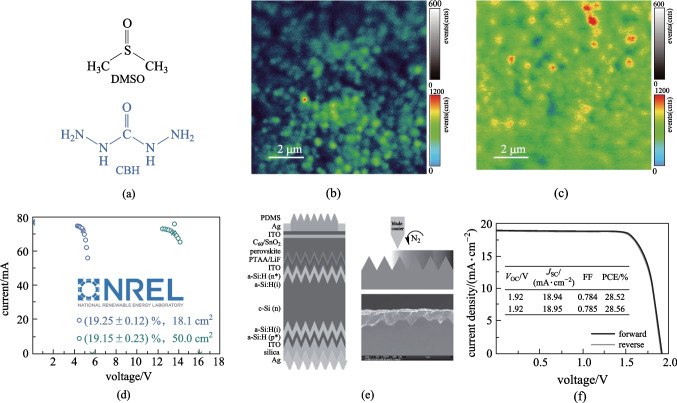
**a** Chemical structures of DMSO and CBH. photoluminescence maps of the control **b** and target **c** perovskite films on thin glass excited from the glass side with a 485-nm laser. **d** NREL certified stabilized current–voltage dots around the MPP point of the minimodules with aperture areas of 18.1 and 50.0 cm^2^. **e** Device structures and cross-sectional SEM images of blade-coated perovskite–silicon tandem cells based on doped-PTAA. **f** *J*–*V* curves of champion monolithic perovskite–silicon tandem solar cell. **a**–**d** Reprinted with permission from Ref. [46]. Copyright 2021, The American Association for the Advancement of Science. **e**, **f** Reprinted with permission from Ref. [48]. Copyright 2022, Springer Nature

In addition to small-area devices, **PTAA** has also achieved remarkable performance when used in large-area devices and tandem devices. Note that due to its non-wetting surface, special composition engineering of perovskites and surface treatment need to be carried out for **PTAA** [46, 47]. For example, Huang et al. partially replaced dimethylsulfoxide (DMSO) in the perovskite precursor solutions with solid-state lead-coordinating additive of carbohydrazide (CBH) (**Fig. ****4****a**) to reduce the interfacial voids [46]. It was found CBH can deposit onto the bottom interface to promote the formation of high-quality perovskite films (Fig. 4b, c), showing reduced charge recombination and facilitated charge extraction. The resulting PVSCs based on blade-coating processed **PTAA** delivered a certified PCE of 19.2% with an aperture area of 50 cm^2^ (Fig. 4d). On this basis, monolithic perovskite-silicon tandem solar cells were further fabricated using **PTAA** doped with 15 wt% 4-isopropyl-4′-methyldiphenyliodonium tetrakis(pentafluorophenyl)borate (TPFB) as the HTL, which realized an encouraging PCE of 28.6% (Fig. 4e, f) [48]. In addition, **PTAA** was also employed to construct monolithic 2-terminal and 4-terminal all-perovskite tandem solar cells by Li et al., enabling high PCEs of 25.15% and 25.05%, respectively (Table 1) [49].

**Table 1:** Device structure and parameters of PVSCs based on distinct polymeric HTMs

HTM	Devices structure	Area/cm^2^	*V*_OC_/V	*J*_SC_/(mA⋅cm^−2^)	FF/%	PCE/%	Ref.
PTAA	ITO/HTL/Cs_0.05_(FA_0.98_MA_0.02_)_0.95_Pb(I_0.98_Br_0.02_)_3_/FcTc_2_/C_60_/BCP/Ag	0.08	1.18	25.7	82.3	25.0	[18]
PTAA	ITO/HTL/MA_0.6_FA_0.4_PbI_3_/C_60_/BCP/Au	50.1	1.15	21.5	79.8	19.7	[46]
PTAA	Ag/Si/ITO/HTL/Cs_0.1_FA_0.2_MA_0.7_Pb(I_0.85_Br_0.15_)_3_/C_60_/SnO_2_/ITO/Ag	1	1.92	19.0	78.5	28.6	[48]
PTAA	ITO/PTAA/FA_0.8_Cs_0.2_Pb(I_0.7_Br_0.3_)_3_/C_60_/SnO_2_/ITO/PEDOT:PSS/(FASnI_3_)_0.6_(MAPbI_3_)_0.4_/C_60_/BCP/Ag	–	1.96	14.6	80.3	25.1	[49]
PPY2	ITO/HTL/(FA_0.92_MA_0.08_)_0.9_Cs_0.1_Pb(I_0.92_Br_0.08_)_3_/C_60_/BCP/Ag	0.11	1.16	23.6	82.0	22.4	[50]
p-PY	ITO/p-PY/Cs_0.05_(FA_0.9_MA_0.1_)_0.95_Pb(I_0.9_Br_0.1_)_3_/C_60_/BCP/Ag	0.09	–	22.6	–	22.4	[51]
p-PY	ITO/p-PY/Cs_0.05_(FA_0.9_MA_0.1_)_0.95_Pb(I_0.9_Br_0.1_)_3_/C_60_/BCP/Ag	1	1.08	23.6	78.7	20.1	[51]
p-PY	ITO/p-PY/Cs_0.15_FA_0.85_Pb(I_0.95_Br_0.05_)_3_/PEAI/PCBM/BCP/Ag	–	1.16	23.8	82.6	22.8	[52]
P3CT-CH_3_NH_2_	ITO/HTL/MAPbI_3_/PC_60_BM/C_60_/BCP/Ag	0.06	1.09	22.2	81	19.6	[56]
P3CT-CH_3_NH_2_	ITO/HTL/(FAPbI_3_)_0.95_(MAPbBr_3_)_0.05_/PCBM/C_60_/TPBi/Cu	0.09	1.19	24.8	82.9	24.3	[57]

**Fig. 5:**
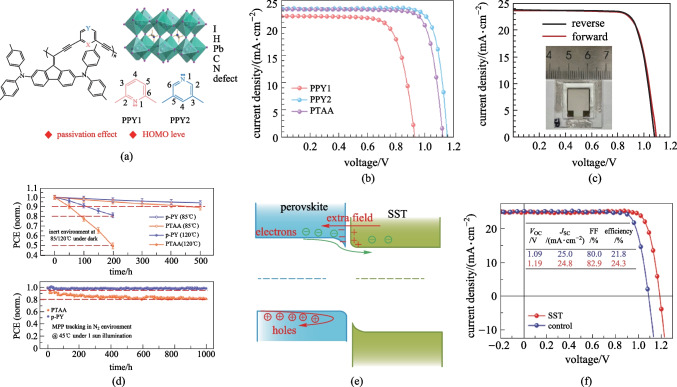
**a** Molecular design strategy of **PPYs**. **b** *J*–*V* curves of the champion PVSCs based on dopant-free **PPY1**-**2** and **PTAA**. **c** Schematic illustration of the device structure based on **p-PY**. **d** Thermal stability of the unencapsulated devices stressed at 120 °C in N_2_ atmosphere in the dark, and light soaking stability of unencapsulated devices under 1 sun equivalent white light LED array illumination with continuous MPP tracking at 45 °C in N_2_ atmosphere. **e** Back-surface field formation at the perovskite surface with surface sulfidation treatment (SST). **f** *J*–*V* curves of control and SST-based PVSCs. **a**, **b** Reprinted with permission from Ref. [50]. Copyright 2021, WILEY–VCH Verlag GmbH & Co. KGaA, Weinheim. **c** Reprinted with permission from Ref. [51]. Copyright 2022, WILEY–VCH Verlag GmbH & Co. KGaA, Weinheim. **d** Reprinted with permission from Ref. [52]. Copyright 2022, Royal Society of Chemistry. **e**, **f** Reprinted with permission from Ref. [57]. Copyright 2022, The American Association for the Advancement of Science

There are also ongoing efforts to develop HTM candidates to replace **PTAA**. Pyridine, a typical Lewis base, is a widely-used passivation group, which can show strong coordination with lead ions. Recently, by utilizing pyridine as the conjugated bridge we successfully designed and synthesized two cross-conjugated polymeric HTMs (**PPY1-2**, Fig. 5a) with high transparency in the visible region and good film forming ability [50]. Through modifying the linkage of pyridine units from 2,6-sites to 3,5-sites, the HOMO level, hole mobility, as well as the passivation ability with lead ions can be effectively optimized, and the resulting **PPY2** with 3,5-linkage showed a reduced HOMO level of − 5.17 eV and an enhanced hole mobility of 1.90 × 10^−3^ cm^2^/(V^−1^⋅s^−1^). Moreover, owing to the strong coordination bonding between pyridine and lead ion, **PPY2** can not only reduce the non-radiative recombination (NRR) at the interface between perovskite and HTL, but also suppress the NRR inside perovskite bulk by facilitating the formation of uniform and highly crystalline perovskite film. As a result, a low *V*_OC_ loss of 0.40 V has been realized for dopant-free **PPY2**-based inverted PVSCs, leading to a high PCE of 22.4% (Fig. 5b). Furthermore, the unencapsulated PVSCs based on **PPY2** HTM showed superior long-term device photostability, with over 97% of initial PCE maintained after 1 sun constant illumination after 500 h.

Recently, Wu et al. also developed a new class of polymer HTMs by simply introducing the pyridine units into **PTAA** side-chains at different substitution positions, and it was shown that para-substituted polymer HTM, denoted as **p-PY** (Fig. 2), showed improved surface wettability, more suitable HOMO level and higher hole mobility, compared to **PTAA**, thereby leading to a high-quality perovskite film and efficient interface hole extraction/transport. The inverted devices based on **p-PY** HTM exhibited a high PCE exceeding 22% and a comparable PCE of ~ 20% for the small area (0.09 cm^2^) and large-area (1 cm^2^) devices, respectively (Fig. 5c) [51]. Later on, Chen et al. further fabricated formamidinium-cesium based inverted PVSCs using **p-PY** as the HTM and realized an enhanced PCE of 22.8% (certified 22.3%) [52]. More importantly, the **p-PY**-based devices showed high long-term stability, retaining 97.5% and 94% of initial PCE after 1000 h under 1 sun light-soaking and 500 h after thermal stress at 85 °C, respectively (Fig. 5d).

Polymer electrolytes such as PEDOT:PSS are another class of important HTMs widely-used in inverted PVSCs due to high conductivity and good wettability with polar solvents [53–55]. However, it is still challenging to achieve high device performance with this type of HTMs. Previously, Fang et al. designed a polythiophene-based polyelectrolyte HTM, **P3CT-CH**_**3**_**NH**_**2**_ (Fig. 2), and achieved a PCE of 19.6% in inverted PVSCs [56]. Based on this HTM, they recently developed an efficient surface sulfidation treatment (SST) to construct stable heterojunctions, and the induced strong Pb–S bonds could not only enable an extra back-surface field for electron extraction but also strengthen underlying perovskite structure (Fig. 5e) [57]. Finally, the SST-derived PVSCs based on **P3CT-CH**_**3**_**NH**_**2**_ HTM delivered an impressive PCE of 24.2% (Fig. 5f), and over 90% of the initial value can be retained after 2200 h at 85 °C.

## SAM HTMs for inverted PVSCs

SAMs are ordered arrays of organic molecules with a thickness of one or few molecules formed by the spontaneous adsorption of the anchoring groups, which are functional groups that can link into the substrate [58]. Unlike common small molecule HTMs, SAM-HTMs can link the substrate via chemical bonding to form a highly stable and extremely thin HTL, which can not only resist damage caused by the perovskite precursor solutions but also provide a robust and smooth interface for growth and crystallization of perovskite [59]. The extremely thin SAM-HTL with efficient hole transfer ability can avoid charge accumulation at the interface of perovskite/HTL, thereby reducing the interface recombination energy losses and also improving the perovskite quality and the phase stability [60]. In addition, the SAM-HTMs with a positive dipole moment can reduce the work function of ITO to promote the hole extraction and transfer [61]. These unique properties have enabled rapid efficiency improvement of SAM-HTMs, with the best PCE exceeding 23.6% achieved already [62]. Besides, SAM-HTMs also exhibit attractive advantages such as low cost, low material consumption and simple fabrication. In this section, we will thus focus on the recent progress of SAM-HTMs for efficient inverted PVSCs, as well as their applications in tandem devices.

**Fig. 6:**
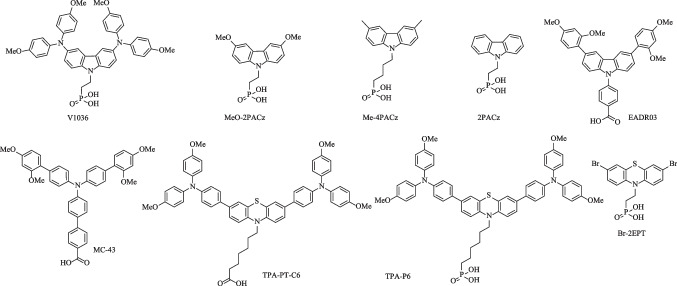
Molecular structure of representative SAM-based HTMs

The structures of representative SAM-HTMs are provided in Fig. 6, with the photovoltaic data summarized in Table 2. In general, a SAM-HTM consists of three parts; (1) an anchoring group that links the HTM molecules to the surface of substrate via chemical bonding; (2) the linkage that connects the anchoring group and functional group; (3) the functional group that directly contacts the perovskite layer [63]. So far, there are two main types of anchoring groups for SAM-HTMs, i.e., phosphonic acid and carboxyl acid. For the carboxyl acid group, two oxygen atoms process two binding sites, and thus allow mono and bidentate binding with the ITO substrate, formed by condensation of hydroxyl groups in the anchoring group with surface hydroxyl groups to form C–O–M bond [64]. The phosphonic acid anchoring group can enable the mono, bidentate and tridentate binding modes with the substrate, leading to much stronger bonding strength compared to that for the carboxyl acid [65, 66]. The types of anchoring groups largely determine the adsorption dynamics, loading density, and bonding strength of the HTM molecules onto substrates, which therefore can significantly affect the device performance and stability. Nowadays, use of the phosphonic acid anchoring group is the preferred choice.

**Table 2:** Device structure and parameters of PVSCs based on distinct SAM-HTMs

HTM	Devices structure	Area/cm^2^	*V*_OC_/V	*J*_SC_/(mA⋅cm^−2^)	FF/%	PCE/%	Ref.
**V1036**	ITO/HTL/Cs_0.05_(MA_0.17_FA_0.83_)_0.95_Pb(I_0.83_Br_0.17_)_3_/C_60_/BCP/Cu	0.16	1.09	21.4	76.5	17.8	[33]
**MeO-2PACz**	ITO/HTL/MA_0.05_FA_0.95_Pb(I_0.95_Br_0.05_)_3_/C_60_/BCP/Cu	0.16	1.12	23.5	80.6	21.2	[61]
**MeO-2PACz**	ITO/HTL/FA_0.8_Cs_0.2_Pb(I_0.5_Br_0.5_)_3_/PC_61_BM/PM6, Y6/Ag	0.02	2.15	14.0	80	24.0	[67]
**Me-4PACz**	Ag/AZO/Si/ITO/HTL/Cs_0.05_(FA_0.77_MA_0.23_)_0.95_Pb(I_0.77_Br_0.23_)_3_/(LiF)/C_60_/SnO_2_/IZO/Ag/LiF	–	1.90	19.26	79.5	29.2	[60]
**2PACz**	IO:H/HTL/(FA_0.8_Cs_0.2_(I_0.6_Br_0.4_)_3_/LiF/C_60_/SnOx/ITO/PDOT:PSS/Csx(FA_0.83_MA_0.17_)_(1−x)_Sn_0.5_Pb_0.5_I_3_/PC_61_BM/BCP/Cu	12.25	13.3	24.8	71	19.1	[69]
**2PACz** + **MeO-2PACz**	PET/ITO/MBNiO/FA_0.8_Cs_0.2_PbI_1.95_Br_1.05_/C_60_/ALDSnO_2_/Au/PEDOT:PSS/FA_0.7_MA_0.3_Pb_0.5_Sn_0.5_I_3_/C_60_/BCP/Cu	0.05	2.00	15.8	78.3	24.7	[70]
**2PACz** + **MeO-2PACz**	PET/ITO/MBNiO/FA_0.8_Cs_0.2_PbI_1.95_Br_1.05_/C_60_/ALDSnO_2_/Au/PEDOT:PSS/FA_0.7_MA_0.3_Pb_0.5_Sn_0.5_I_3_/C_60_/BCP/Cu	1.05	2.02	15.7	74.1	23.5	[70]
**EADR03**	ITO/HTL/Cs_0.05_FA_0.79_MA_0.16_Pb(I_0.84_Br_0.16_)_3_/C_60_/BCP/Cu	–	1.16	22.9	80	21.2	[71]
**MC-43**	ITO/HTL/MAPbI_3_/PC_60_BM/Ag	0.09	1.07	20.3	80	17.3	[59]
**TPA-PT-C6**	ITO/HTL/MAPbI_3_/PC_61_BM/BCP/Ag	1.02	–	–	–	17.5	[72]
**TPT-P6**	ITO/HTL/Cs_0.05_MA_0.12_FA_0.83_Pb(I_0.85_Br_0.15_)_3_/C_60_/BCP/Ag	0.09	1.13	–	81.1	21.4	[73]
**Br-2EPT**	FTO/HTL/Cs_0.05_(FA_0.92_MA_0.08_)_0.95_Pb(I_0.92_Br_0.08_)/C_60_/BCP/Cu	0.11	1.09	25.11	82	22.4	[74]

In addition, inactive aliphatic linkage and photoactive conjugated unit also play critical roles. The linkage can not only induce different molecular packing and geometry with Van der Waals interactions, but also provide a well-defined thickness with specific size and tilt angle and acts either as tunneling for the free-carriers or as physical barriers, both of which influence the charge transport properties at the interface. The aromatic units as well as their functional groups can form a new surface with perovskites, serving as a growth template for the overlying perovskite layer in addition to extracting/transporting the holes. So far, the most commonly used aromatics in SAM-HTMs, as shown in Fig. 6, are typical electron-rich units, including carbazole, triphenylamine and phenothiazine, etc.

Two fabrication methods have been used for SAM-HTMs, namely dip-coating and spin-coating. For the dip-coating method, the substrate is immersed into the SAM-HTM solution where the molecules chemically adsorb. The process can be controlled by adjusting the solvent, concentration of HTMs and dipping time. For the spin-coating method, a uniform film is produced by dispensing the SAM-HTM solution onto the substrate surface at a certain speed. For both methods, the surplus unbonded molecules are later removed by solvent washing after a thermal annealing process that can strengthen the bonding between SAM-HTMs and the substrate.

**Fig. 7:**
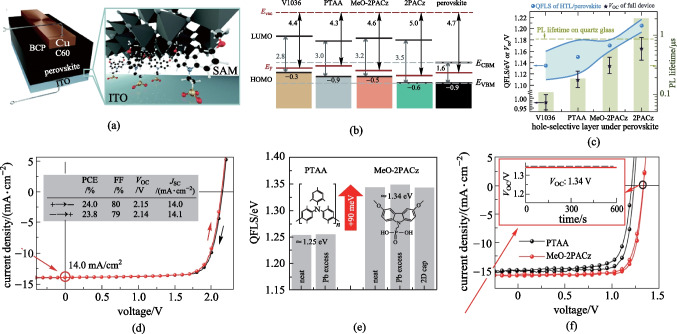
**a** Schematic of the investigated device structure based on **MeO-2PACz**. **b** Schematic representation of the band edge positions of the investigated HTMs based on values from ultraviolet photoelectron spectroscopy (UPS), referenced to the vacuum level. **c** Summary of abs photoluminescence and time-resolution photoluminescence measurements. **d** *J*–*V* characteristics of the champion perovskite-organic tandem cells based on MeO-2PACz. **e** Splitting of the quasi-Fermi-level splitting (QFLS) in the case of varied HTMs. **f** *J*–*V* characteristics for champion wide-gap perovskite subcells with a 2D capping layer. **a**–**c** Reprinted with permission from Ref. [61]. Copyright 2019, Royal Society of Chemistry. **d**–**f** Reprinted with permission from Ref. [67]. Copyright 2022, Springer Nature

Carbazole possesses many advantages such as excellent hole-transporting capability, rigid backbone and easy functionalization, thereby making it an ideal platform for SAM-HTMs. Indeed, the first SAM-HTM for inverted PVSC was reported using carbazole as the building block in 2018 by Getautis et al., namely **V1036** (Fig. 6). Phosphonic acid was used as the anchoring group, with ethyl group as the linkage and dimethoxydiphenylamine units as the functional groups [33]. However, due to the poor interface contact caused by the large size of functional groups, it was necessary to add small electrically filler molecule, i.e., butylphosphonic acid (C4), to optimize the ionization potential and wettability. The resulting PVSCs based on the mixture of a 10% **V1036** and 90% C4 achieved a PCE of 17.8% with a high FF of 81.0% (Table 2). Subsequently, Albrecht et al. further designed and synthesized a new carbazole-based SAM-HTM, **MeO-2PACz**, through a much simpler structural design, in which methoxy was used as the functional group of carbazole (Fig. 7a) [61]. The **MeO-2PACz** not only provided a well-suited energy level but also strongly reduced the NRR at the interface (Fig. 7b, c). Benefiting from the small size of methoxy group, no filler molecules were required for constructing the SAM-HTM, and the resulting inverted MeO-2PACz-based PVSCs delivered a much-enhanced PCE of 21.1%. Since then, SAM-HTMs have attracted more interest.

**Fig. 8:**
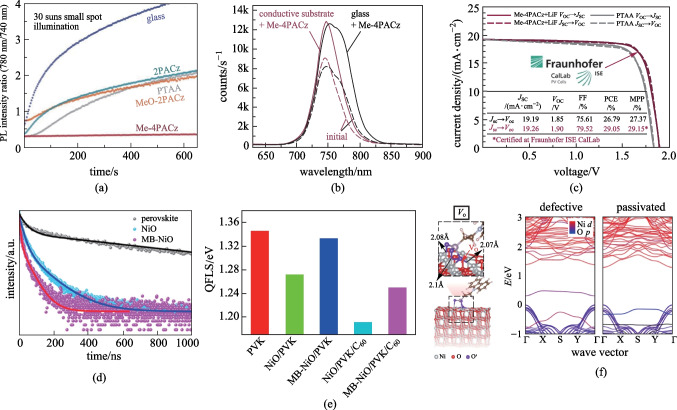
**a** Ratio of PL intensities at 780 nm (I-rich domains) and 740 nm (neat perovskite) during PL evolutions at high illumination intensity. **b** PL spectra before (dashed lines) and after 600 s of light-soaking (solid lines) under 1-sun equivalent illumination in air, comparing the perovskite grown on **Me-4PACz** that had been deposited on a glass substrate and a conductive ITO substrate. **c** Certified *J*–*V* curve measured at Fraunhofer ISE, including the MPP value and the device parameters (red), in comparison to a tandem cell with **PTAA** (gray) as HTL measured in-house. **d** TRPL spectra of perovskite films deposited on PET, PET/ITO/NiO and PET/ITO/MB-NiO substrates. **e** Calculated QFLS of the perovskite film, HTL/perovskite and HTL/perovskite/electron transport layer junctions. **f** First-principles simulation of passivation effect of the phosphoryl group in the bridging molecule (that is, **2PACz**) on typical defects on NiO (O vacancy: VO (**e**)) surfaces. **a**–**c** Reprinted with permission from Ref. [60]. Copyright 2021, The American Association for the Advancement of Science. **d**–**f** Reprinted with permission from Ref. [70]. Copyright 2022, Springer Nature

More encouragingly, further study demonstrated that inverted PVSCs based on carbazole-based SAM-HTMs have shown high compatibility for tandem devices. In 2022, a **MeO-2PACz**-based SAM-HTL was first utilized by Riedl et al. to construct perovskite-organic tandem cells, showing a high PCE of 24.0% (certified 23.1%) and a high *V*_OC_ of 2.15 V (Fig. 7d) [67]. As shown in Fig. 7e, f, **MeO-2PACz** led to a larger quasi-Fermi-level splitting (QFLS) compared to PTAA, thereby offering a very high stabilized *V*_OC_ of 1.34 V for the perovskite subcell. Moreover, Albrecht et al. also successfully fabricated the monolithic perovskite/silicon tandem solar cells utilizing **Me-4PACz** as the SAM-HTM (Fig. 6), containing the butyl group as the linkage and two methyl functional groups in the carbazole [60]. The **Me-4PACZ** was found to simultaneously achieve fast hole extraction and efficient passivation at the hole-selective interface (Fig. 8a, b), producing a high *V*_OC_ over 1.23 V in single-junction device. As a result, the monolithic perovskite/silicon tandem solar cell based on **Me-4PACz** realized a record certified PCE of 29.2% (Fig. 8c).

In addition, Paetzold et al. found that **2PACz** (Fig. 6) without any functional groups in the carbazole can be used as an efficient SAM-HTM [68], and further fabricated the laser-scribed all-perovskite tandem modules via blade coating and vacuum deposition based on **2PACz**, delivering a PCE of 19.1% with an aperture area of 12.25 cm^2^. The **2PACz** with a large molecular dipole moment of 2.0 Debye can reduce the work function of ITO to increase the *V*_OC_ [69]. Furthermore, Tan et al. employed a mixture of **2PACz** and **MeO-2PACz** with significantly different dipole moments of 2.0 D and 0.2 D, respectively, as the molecule-bridged interface anchoring on the low temperature-processed NiO nanocrystal film [70], and this can finely tune the energy-level alignment between NiO and wide-bandgap (WBG) perovskites to mitigate the interfacial recombination (Fig. 8d–f). Using this optimized molecule-bridged-NiO as the HTL of WBG front subcell, the fabricated flexible all-perovskite tandem solar cells achieved high PCEs of 24.7% and 23.5% for the small area (0.05 cm^2^) and large area (1.05 cm^2^) devices, respectively. Furthermore, Palomares et al. developed an atypical SAM-based HTM, **EADR03** (Fig. 6), using carboxylic acid as the anchoring group, phenyl ring as the linkage and 1,3-dimethoxybenzene as the functional group [71]. The resulting devices based on **EADR03** delivered a high PCE of 21.2%, demonstrating the structural diversity of carbazole-based SAM-HTMs.

**Fig. 9:**
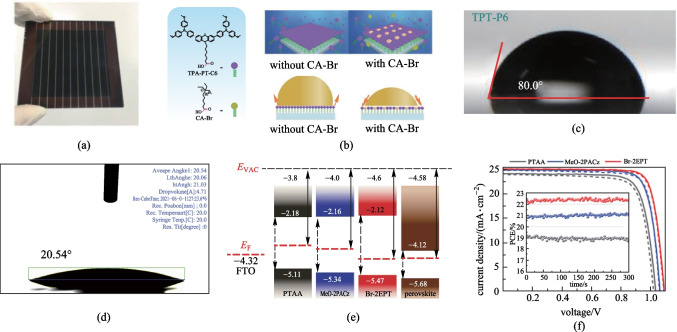
**a** Photograph of a small module based on **TPA-PT-C6**. **b** Molecular structures of the **TPA-PT-C6** and co-adsorbent CA-Br, and the deposition process of the self-assembly HELs with and without co-adsorbent. **c** Water contact angle measurements of **TPT-P6** after solvent wash. **d** Contact angle measurements FTO/**Br-2EPT**. **e** Energetic alignment of different HTL contacts and CsMAFA perovskite with reference to vacuum level, where the dashed red lines are the Fermi energy levels measured with UPS. **f** *J*–*V* curves of the best-performing PVSCs under simulated AM 1.5G illumination at a scan rate of 100 mV/s in forward (*J*_SC_ to *V*_OC_, dashed) and reverse scan (*V*_OC_ to *J*_SC_, solid) with respective MPP tracks in the inset. **a**, **b** Reprinted with permission from Ref. [72]. Copyright 2020, WILEY–VCH Verlag GmbH & Co. KGaA, Weinheim. **c** Reprinted with permission from Ref. [73]. Copyright 2021, WILEY–VCH Verlag GmbH & Co. KGaA, Weinheim. **d**–**f** Reprinted with permission from Ref. [74]. Copyright 2022, WILEY–VCH Verlag GmbH & Co. KGaA, Weinheim

Besides carbazole, diphenylamine and phenothiazine with low ionization potential have been also successfully used for SAM-based HTMs (Fig. 6). However, their performance needs to be further improved. Palomares et al. designed a SAM-HTM, **MC-43** (Fig. 6), utilizing diphenylamine derivative as the functional group and biphenyl ring as the rigid linkage [59]. The inverted PVSCs based on **MC-43** showed a moderate PCE of 17.3%. In 2020, Wu et al. designed a new SAM-HTM, **TPA-PT-C6** (Fig. 6), by using carboxylic acid as the anchoring group, hexyl group as the linkage, and triphenylamine derivative as the functional group of phenothiazine, and a PCE of 17.5% was obtained in large-area devices with an aperture area of 1.02 cm^2^ (Fig. 9a) [72]. Subsequently, they developed **TPT-P6 **(Fig. 6) by using phosphonic acid as the anchoring group instead of carboxylic acid to realize a high PCE of 21.43% based on small-area devices [73]. It is worth mentioning that both **TPA-PT-C6** and **TPA-P6** needed to be used with additional filler molecules or interface layer to regulate the wettability of SAM-HTL with perovskite precursor solutions (Fig. 9b, c), due to the use of bulky and hydrophobic triphenylamine-substituents and long hexyl bridge. Recently, Hong and coworkers also reported another efficient SAM-HTM, **Br-2EPT** (Fig. 6), by using phosphonic acid as the anchoring group, ethyl group as the linkage, and bromine atom as the functional group of phenothiazine [74]. The short linkage and small size of functional group reduced the hydrophobicity of **Br-2EPT** and thus led to a small water contact angle of 20.54° and a decreased HOMO level (Fig. 9d, e). The resulting inverted PVSCs based on **Br-2EPT**-based SAM-HTL finally achieved a high PCE of 22.44% (certified 21.81%) without filler molecules and interface layer (Fig. 9f). Moreover, the devices also showed outstanding operational stability without any PCE loss after 100 h of continuous MPP tracking.

## Conclusion and outlook

Inverted PVSCs have achieved significant progress in recent years, and now their record PCE has exceeded 25%, which narrows the efficiency gap with conventional PVSCs. With the continuous breakthroughs made in tandem devices, inverted PVSCs have huge commercialization potential and will become a hot spot in photovoltaic research. The encouraging development of organic HTMs indeed plays an indispensable role in enhancing the performance of inverted PVSCs, and conjugated polymer and SAM represent two avenues for the future development of high-performance HTMs toward efficient and stable inverted PVSCs. In this review, we have summarized the encouraging progress of polymer and SAM-based HTMs achieved in the past three years, mainly focusing on new molecular design strategies, device performance, and the emerging application in tandem solar cells.

Initially, polymer HTMs led the development of inverted PVSCs. Owing to the abundant polymerization monomers and structural optimization strategies including main-chain and side-chain engineering, the solution processability, photophysical and optoelectronic properties of polymer HTMs, such as transmittance, energy level, hole mobility and green-solvent processability, can be simply and precisely regulated. However, polymer HTMs still face several problems before their commercialization becomes feasible. The first one is their high cost and poor batch-to-batch repeatability, due to the reliance on high-cost palladium catalysts and strict reaction conditions [75, 76]. Indeed, this is a general and difficult challenge for semiconducting polymer materials. Controlled free-radical polymerization of vinyl monomers is a feasible solution for constructing low cost HTMs with good reproducibility, which however is limited by a scarcity of suitable monomers and thus the PCE development remains tardy. In addition, most inverted PVSCs based on polymer HTMs still have a large voltage loss that limits their device performance. Molecular design strategies that can provide suitable passivation functions are much needed not only for suppressing the interface NRR but also for facilitating the growth of overlying perovskite films. For example, introducing a small amount of polar or ionic groups into the hydrophobic polymeric HTMs is a feasible strategy that can promote the uniform dispersion of the perovskite precursor and the formation of perovskite polycrystalline films with large grains.

Although the first SAM-HTM for inverted PVSCs was only reported in 2018 [33], it has been considered to be an alternative to the polymer HTM for inverted PVSCs due to the advantages of low cost, no reproducibility issue, easy fabrication, high interface stability and excellent adaptability to tandem devices. However, compared to the polymer HTMs, the variety of SAM-HTMs is still insufficient, and so far the molecular structures of high-performance SAM-HTMs have been mainly focused on fixed combinations of functionalized carbazole unit with phosphate anchoring group. Exploring new core building blocks will be important for expanding the variety of SAM-HTMs, thereby offering more choices for perovskites with different compositions as well as different type of devices. Another challenge is the lack of anchoring groups in most SAM-based HTMs to bond perovskites, meaning that design of double-anchored functional SAM-HTMs is to be preferred. This design could not only provide a good contact to reduce the interface energy loss, but also promote the crystallization of perovskites, thereby further improving the device efficiency and stability. In this context, rational molecular design strategies toward double-anchored functional SAM-HTMs will be highly desirable in the future.

For both types of HTMs, one common and significant challenge is how to design them to be suitable for the fabrication of large-area inverted PVSCs. Spin coating is an ideal processing method for small-area devices, but is not suitable for large-area device fabrication [77, 78]. In this regard, it is important to pay more attention to developing HTMs that can be compatible with advanced large-area film processing methods such as blade coating and roll-to-roll printing, because the crystallization process of perovskite films is quite different when changing the film processing methods. Forming high-quality large-area perovskite films overlying the HTL is still a challenging task in the field. In this context, the effects of HTMs on the growth and crystallization of perovskites fabricated by large-area film processing methods have to be systematically studied through tuning their molecular crystallinity, film surface properties and interactions with perovskites, etc. In this way, the design rules of HTMs for large-area PVSCs can be clarified. Overall, we believe that with the help of new developments in HTMs, the performance of inverted PVSCs will make further exciting progress in the near future.
